# Service-Centric Heterogeneous Vehicular Network Modeling for Connected Traffic Environments

**DOI:** 10.3390/s22031247

**Published:** 2022-02-07

**Authors:** Ahmad M. Khasawneh, Mamoun Abu Helou, Aanchal Khatri, Geetika Aggarwal, Omprakash Kaiwartya, Maryam Altalhi, Waheeb Abu-ulbeh, Rabah AlShboul

**Affiliations:** 1 Department of Cybersecurity, Amman Arab University, Amman 11953, Jordan; a.khasawneh@aau.edu.jo; 2 Faculty of Information Technology, Al Istiqlal University, Jericho 4728, Palestine; mabuhelou@pass.ps (M.A.H.); w.abuulbeh@pass.ps (W.A.-u.); 3 Department of Computer Science, Sat Jinda Kalyana PG College, Kalanaur 124113, Haryana, India; aancha84_scs@jnu.ac.in; 4 School of Science and Technology, Nottingham Trent University, Nottingham NG11 8NS, UK; geetika.aggarwal@ntu.ac.uk; 5 Department of Management Information System, College of Business Administration, Taif University, P.O. BOX 11.99, Taif 21944, Saudi Arabia; marem.m@tu.edu.sa; 6 Computer Science Department, Faculty of Information Technology, Al al-Bayt University, Mafraq 25113, Jordan; rabahshboul@aabu.edu.jo

**Keywords:** heterogeneous vehicular communication, Internet of connected vehicles, vehicular ad hoc networks, heterogeneous networking, Internet of Things

## Abstract

Heterogeneous vehicular communication on the Internet of connected vehicle (IoV) environment is an emerging research theme toward achieving smart transportation. It is an evolution of the existing vehicular ad hoc network architecture due to the increasingly heterogeneous nature of the various existing networks in road traffic environments that need to be integrated. The existing literature on vehicular communication is lacking in the area of network optimization for heterogeneous network environments. In this context, this paper proposes a heterogeneous network model for IoV and service-oriented network optimization. The network model focuses on three key networking entities: vehicular cloud, heterogeneous communication, and smart use cases as clients. Most traffic-related data–oriented computations are performed at cloud servers for making intelligent decisions. The connection component enables handoff-centric network communication in heterogeneous vehicular environments. The use-case-oriented smart traffic services are implemented as clients for the network model. The model is tested for various service-oriented metrics in heterogeneous vehicular communication environments with the aim of affirming several service benefits. Future challenges and issues in heterogeneous IoV environments are also highlighted.

## 1. Introduction

A universal network architecture is being envisioned considering the significant growth in sensor-enabled digital things in our daily life such as smartphones in our hands, vehicles on roads, entertainment devices in homes, and computing systems in offices [1]. This global network architecture leverages most of the existing networks. It is adopted as the Internet of things (IoT) in academic and industrial research communities. Interoperability is the key feature for achieving seamless integration of heterogeneous networks by utilizing intelligent interfaces [2]. The Internet of connected vehicles (IoV) is a heterogeneous network that has evolved from the existing ad hoc network–oriented vehicular communication. It integrates different vehicular networks in road traffic environments (i.e., vehicle-to-vehicle (V2V), vehicle-to–roadside unit (V2R), vehicle-to–in vehicle sensors (V2S), vehicle-to–mobile infrastructure (V2I), and vehicle-to–personal device (V2P)-enabled vehicular networks) [3,4]. The ad hoc network-oriented conventional vehicular network aimed to enhance traffic safety and efficiency via real-time communication between on-road vehicles utilizing roadside units. Various standards and protocols have been developed to enable ad hoc vehicular networks, including wireless access in vehicular environments (WAVE) and dedicated short-range communication (DSRC) [5]. 

Ad hoc network-enabled vehicular communication technology lacks commercial interest toward implementation, despite the lower operational cost–driven networks for traffic safety and efficiency services [6]. This is due to the ad hoc vehicular networks’ inability to operate compatibly with existing heterogeneous network technologies [7]. Specifically, the issues include pure ad hoc communication architecture, lack of standards for personal devices, intermittent Internet service, and cooperative dependency for network operations. The pure ad hoc network architecture cannot support service-oriented commercial applications [8]. The intelligent decisions based on the enormous amounts of traffic data are far from reality due to the unavailability of cloud support in intermittent Internet service [9]. The growing number of personal devices are dead-ends for vehicles. The compatibility is still a serious challenge considering the heterogeneous personal devices prevalent in traffic environments [10]. Due to cooperative information processing, time-constrained traffic information delivery is far from reality [11]. Moreover, the ever growing connected world era has significantly affected ad hoc vehicular communication in terms of the futuristic, connected-vehicle framework. A vehicle would always remain connected to the Internet via smart-handover-enabled heterogeneous reachable networks. 

This paper proposes a heterogeneous network model for enabling the IoV framework. A practice-oriented modeling approach is followed to design and develop the framework. It has significant potential to enable the connected-vehicle paradigm and to spur commercial interest in vehicular communication. Specifically, we aim to answer the following questions:

- What are the key technical components involved in realizing a heterogeneous vehicular network model for the IoV?
- How to realize vehicular cloud-oriented data processing in vehicular environments enabling big traffic data computation for making intelligent traffic decisions?
- How to perform heterogeneous connection management and prioritization in dynamic vehicular traffic environments?
- Is the provisioned heterogeneous vehicular network model for the IoV efficient and scalable considering the growing network heterogeneousness, vehicle speed, and density?

The rest of the paper is articulated as follows: In Section 2, a critical review of the related literature is carried out. Section 3 presents the details of the proposed heterogeneous network model. Section 4 discusses the service-oriented performance evaluation of the network model, followed by the conclusions presented in Section 5.

## 2. Related Work

Research and development on the connected-vehicle-traffic environment are gaining momentum in the past few years due to the growing government-level support in this the area in most developed countries, particularly in the UK, US, and EU countries [12]. An integrated vehicular network name, i.e., Space–Air–Ground (SAGiven) has been suggested, focusing on heterogeneous network function and network resource identification [13]. It has developed a vehicular communication framework considering mobile-network-connected on-road ground vehicles, unmanned aerial vehicles (UAVs), and satellites-based space vehicles. The framework uses a case-based study rather than a scientific novelty as no new technologies or concepts have developed; instead, existing techniques have been utilized. Another UAV-enabled connected-vehicle framework was investigated, focusing on 6G communication-centric services [14]. A UAV-centric task-offloading technique was developed for edge devices in a vehicular network environment considering the high computing capacity in the 6G communication environment. The edge devices that communicate with a particular UAV were identified as an edge network group. However, the focus of this study was on reducing the energy consumption of edge devices in a vehicular network. The issue of energy consumption is not a potential issue in the vehicular network considering vehicles’ battery capacity. A similar UAV-based content distribution vehicular network has been suggested considering 5G-centric IoT services [15]. Initially, an integrated network architecture was developed to optimize the quality of experience (QoE) for vehicle drivers. The integrated network involves a UAV network and a vehicular network for traffic-related content distribution. These UAV-based vehicular network integration frameworks have considered only mobile network integration without focusing on other personal networks’ integration with vehicular networks.

The other dimension of research on enabling the Internet of connected vehicle environment is improving the performance of heterogeneous network architecture using innovative techniques [16,17]. A cooperative driving framework has been suggested for the Internet of connected vehicle environment, focusing on velocity prediction of neighboring-vehicle-centric motion planning for path following a driving scenario [18]. The driving data of nearby vehicles were utilized in a neural-network-based framework for generating a safe travel pattern considering the predicted velocity error of all the neighboring drivers. It was validated for lane-changing scenarios in the connected vehicle environment. However, the framework relies on the precision and accuracy of the driving data of neighboring vehicles.

Similarly, another cooperative driving control framework was investigated for the Internet of connected vehicle environment, focusing on collision avoidance at merging roads [19]. The merging road area was divided into three subregions, including delay estimation region, control region, and merging region, for precisely calculating communication delay considering the dynamic mobility data of approaching vehicles and other traffic data in the region. The study assumed roadside infrastructure-based communication, which has practically difficult deployment limitations near all the merging roads. Another cooperative collision avoidance framework has been explored considering trajectory prediction and mobility uncertainty in connected vehicle environments [20]. An edge and cloud server–based vehicle-to-roadside unit reliable communication architecture was considered for improving traffic-data-centric knowledge on mobility uncertainty. The cooperative collision avoidance framework relies on roadside-infrastructure-based communication rather than a connected-vehicle networking environment. The aforementioned studies in the connected-vehicle environment majorly focused on network performance improvement rather than on network prioritization in the heterogeneous vehicular network environment, which is the scope and focus of this paper. 

## 3. Internet of Connected Vehicles

### 3.1. Heterogeneous Vehicular Networks

The IoV is a global vehicular network leveraging the Internet and various vehicular networks in traffic environments. The proposed heterogeneous vehicular network architecture leverages different kinds of vehicle-oriented networking, including V2V, V2R, vehicle-to–personal devices (V2P), vehicle-to–mobile infrastructure (V2I), and vehicle-to-sensors (V2S) for on-road traffic environments. These vehicular communications are different due to their enabling wireless access technologies. The intervehicle ad hoc type of communication, including V2V and V2R, is supported by WAVE. The long-range V2I web communication is enabled by Wi-Fi and 4G/LTE technologies. The in-vehicle V2P and V2S communications utilize Car-Play and Wi-Fi, respectively. The range of technologies and devices increases the design complexity of the architecture. It is complemented as a market-oriented vehicular communication technology. 

**Figure 1:**
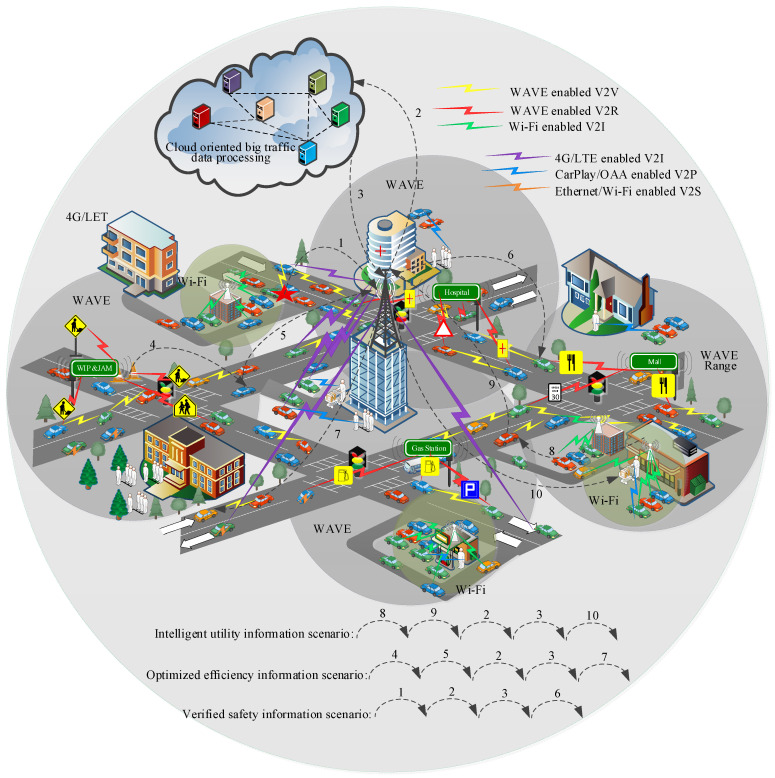
The realization of the IoV scenario with heterogeneous vehicular networks.

The global vehicular network framework has enormous potential not only to guide (with respect to traffic safety and efficiency-related cooperative information sharing among on road vehicles) vehicles but also to supervise (with respect to vehicle-safety-related dedicated information delivery between an intelligent cloud server and vehicles or drivers of vehicles). The abundant traffic applications related to mobile Internet and multimedia services are considered deployable on the heterogeneous vehicular framework using publish–subscribe based architecture. A realistic framework is illustrated in Figure 1, focusing on three advanced traffic information processing scenarios. Firstly, IoV enables the verification of traffic safety information via network coordination. The verified safety information is published over local networks by authorities on global networks. Secondly, the efficiency information available over IoV is near-optimal information, considering the utilization of global traffic scenarios of more significant geographical regions. Thirdly, IoV-based utility information is intelligent due to considering the cloud-oriented market of big-data processing by third-party utility information service providers.

### 3.2. Network Model

**Figure 2:**
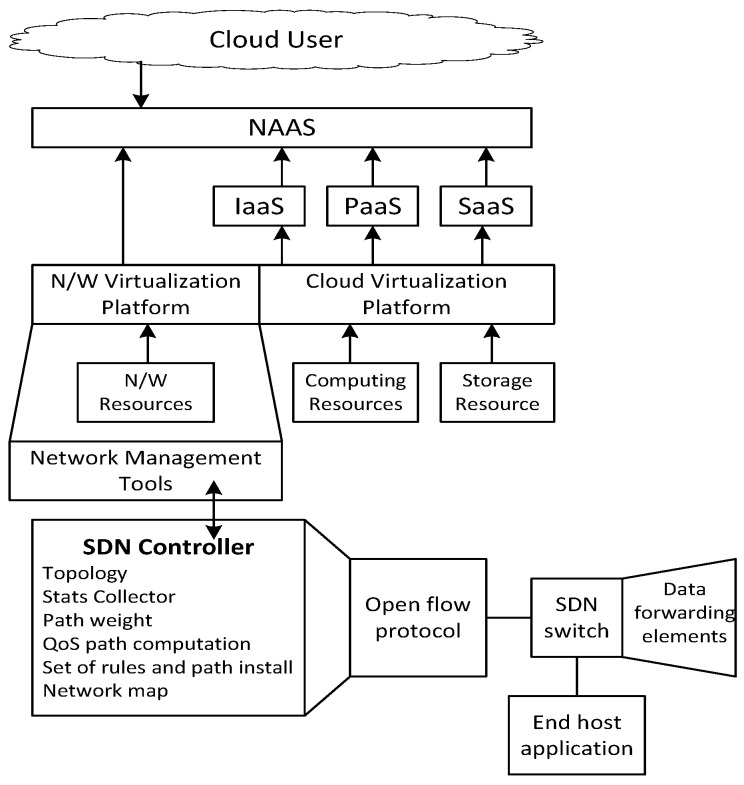
System architecture.

The network model of IoV is based on cloud-oriented big traffic data computing and heterogeneous-communication-oriented intelligence. The proposed model considers the concept of graph partitioning in order to achieve quality of service (QoS) flow allocation and prioritization for multitenants. Here, QoS means traffic-service-centric resource allocation in the integrated vehicular network environment. Dijkstra’s and Kruskal’s algorithms were used to model the procedure of multitenancy QoS path computation. The concept of multitenancy allows sharing of resources and applications. However, its drawback is that some dominating tenants can monopolize the resources, which results in system performance degradation. Therefore, the concept of a software defined network (SDN) was used to overcome this problem. In general, SDN is a network virtualization concept for enabling specific service-centric networks. Here, SDN was used to control the tenants and the network usage in vehicular network integration. The network management layer performed network virtualization, which resulted in the separation of different tenants’ flows to increase isolation among tenants. After that, different flows were controlled by SDN dynamically. The SDN handled the virtual network layers and stored subnets of the physical network. SDN based system overview is presented in Figure 2. 

The network controller requested a management tool to assign the best-suited route whenever new flow and resources arrived in the network. The network management tools allocated the best resources to newly arrived flow based on the current status of the network. The concept of graph partitioning was used to achieve QoS flow allocation and prioritization for multitenants. The symbols used in this paper are summarized in Table 1.

**Figure 3:**
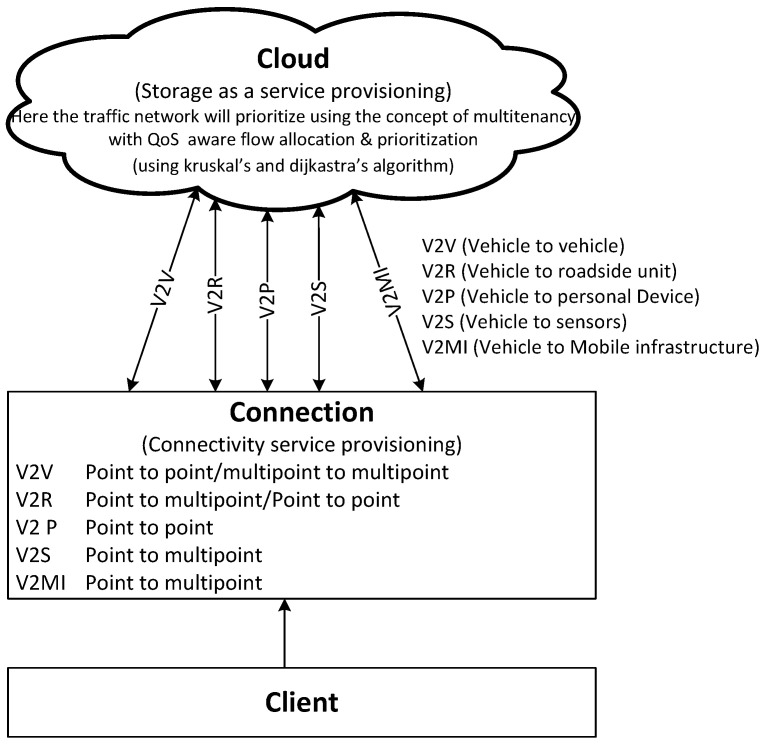
Building blocks of the network model.

**Figure 4:**
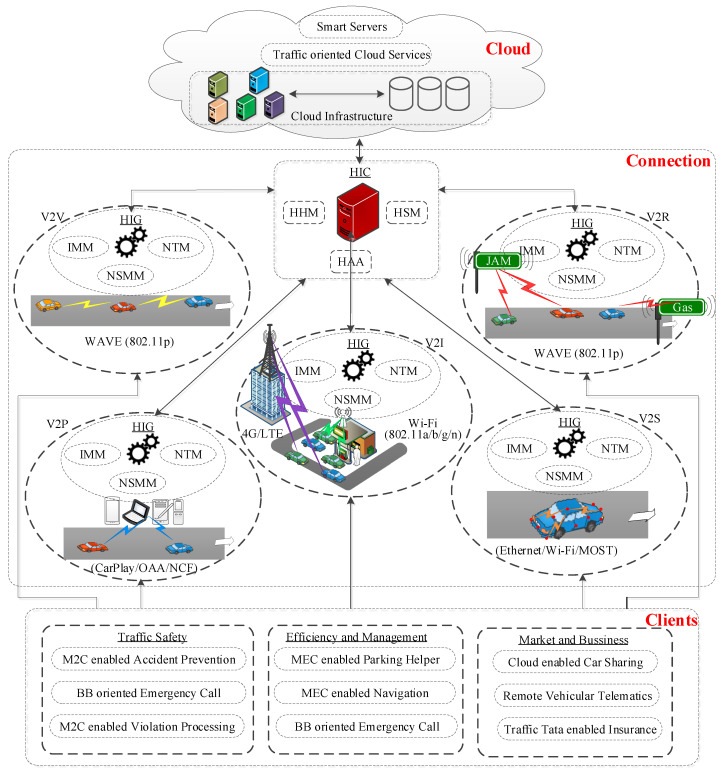
Vehicular cloud-oriented heterogeneous network model for IoV.

The building blocks of the network model of the IoV include the cloud, the connection, and the clients as key network components (Figure 3). The cloud represents the computing brain, enabling unlimited processing capability in vehicular environments. The cloud-based services are accessible via a reliable vehicle-to-Internet connection. The vehicle-oriented connection is a cooperative combination of various wireless access technologies with vehicular networks. Various traffic-utility-oriented clients utilize the heterogeneous network access technology–enabled Internet connection for making intelligent decisions via cloud-based computing resources. The inner-module-oriented relational structure of the proposed heterogeneous vehicular network model is presented, focusing on key network entities (see Figure 4).

(1) Vehicular Cloud

The cloud framework has two key operation levels for realizing cloud-oriented intelligent application servers. The traffic-oriented essential cloud services are considered lower-level, whereas smart IoV servers are developed on top of these essential services. The distinguishable level-wise cloud operation is significant considering the steep growth in the volume of traffic data once the integration of various existing networks with the vehicular network is realized as an IoV. The traffic data uploading, information processing, dissemination, and storage are the basis for the two-level cloud operation on big traffic data. The technical roles of each level of operations are introduced below:

- Traffic-Oriented Cloud Services

The traffic-oriented cloud services are essential to the intelligence processing and analysis of big traffic data (see Figure 3). The implemented services include Computing as a Service (COaaS) on traffic data, Storage as a Service (StaaS) for traffic data, Data as a Service (DaaS) for traffic information re-utilization, Gateway as a Service (GaaS) for heterogeneous network support, and geo-Location as a Service (LoaaS) for vehicle localization. Some other services toward multimedia intelligence are also implementable, including Picture as a Service (PcaaS) for sharing traffic incidence, Platform as a Service (PlaaS) for system-oriented traffic applications, Software as a Service (SoaaS) for traffic analysis, and Network as a Service (NaaS).

- Smart Server

**Figure 5:**
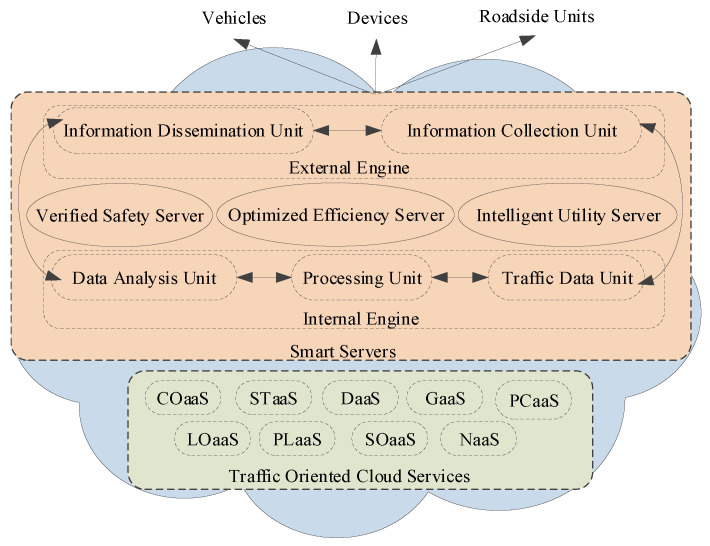
The two-level vehicular cloud engine for IoV.

The smart IoV servers consist of two processing engines, namely, internal and external (see Figure 5). These processing engines utilize traffic-oriented cloud services to infer intelligent decisions from traffic data. The responsibilities of an internal processing engine include materializing big traffic data, processing via applying artificial intelligence, and analyzing with a focus on smartness. The external processing engine is majorly responsible for traffic-oriented data collection and dissemination. The coordination between engines to simulate intelligence enables three types of smart IoV servers: verified traffic safety, optimized efficiency, and intelligent utility toward business-oriented servers. The visualization of three IoV services is due to these smart servers’ different processing and time-oriented constraints.

(2) Connection for Heterogeneous Vehicular Communication

The vehicular connection between smart IoV server and the vehicular end-user is composed of a third-party heterogeneous internetwork coordinator (HIC) and heterogeneous internetworking gateway (HIG). It is operational cooperation between the cloud server and IoV end-user, including vehicles, personal devices, and roadside infrastructure. The coordination-oriented network management in heterogeneous environments, including 802.11p, Wi-Fi, and 4G/LTE access technology, is the key responsibility of the HIC. The HIG represents the individual network connection. The HIC prioritizes network connections based on wireless access technologies.

- Heterogeneous Internetworking Coordinator (HIC)

**Figure 6:**
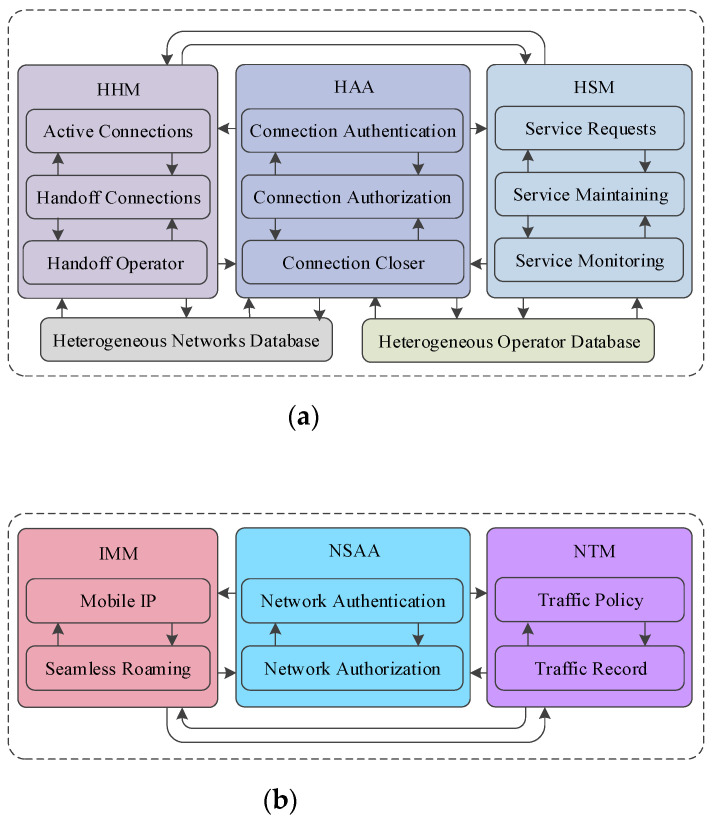
Key functional modules in heterogeneous connection: (**a**) HIC and (**b**) HIG.

The internetwork operator provides a service level agreement to the end-user for the heterogeneous network operators in the IoV. This enables seamless roaming between the heterogeneous networks, enabling internetwork cooperation without compromising the quality of network performance. The HIC eliminates the requirement of a pairwise quality of service agreement between network operators, which is a significantly challenging constraint for any heterogeneous network framework. Three key functional modules were developed in the HIC to carry out the internetworking-oriented connection and service management. The modules include heterogeneous handoff management (HHM), heterogeneous authentication and authorization (HAA), and heterogeneous service management (HSM). These modules interact with two major databases, including heterogeneous network and network operator databases. The functional relationship among key functional modules and databases is presented in Figure 6a. 

The HHM module monitors network connections, looking for potential active connections that may require a internetwork handoff shortly. These active connections transform into handoff connections after the operation confirmation from HAA and HSM. The transformation is enabled by a handoff operator responsible for verifying response confirmation from the authentication and service modules. The HAA module maintains end-user credentials across networks for verification. This includes network- and operator-specific access right validation, bypassing the response for initiating handoff operation. It monitors the session-wise network operation and initiates a time-oriented connection closer in case of an idle connection. The third-party-oriented HIC implementation enables smooth end-user authentication and authorization, which is challenging considering heterogeneous network environments. 

The HSM module provides an end-user service layer agreement using a service quality rating approach for different operating networks. It uses a list of dedicated services between heterogeneous operating networks. The service quality is rated, with a focus on guarantying service quality to end-users by maintaining a service delivery history for each heterogeneous operating network. The rating is implemented considering user-feedback-oriented service monitoring for the connections initiated in the networks. The heterogeneous network and operator database are accessed as a key information resource in the connection-oriented operations of the three functional modules in HIC.

- Heterogeneous Internetworking Gateway

The heterogeneous internetworking gateway of a connection implements network access technologies in IoV to enable effective collaboration with the HIC for initiation and maintenance of heterogeneous vehicular connections. The HIG represents four types of wireless access technologies enabling the five types of vehicular communications. It includes WAVE-enabled V2V and V2I, Wi-Fi- or 4G/LTE-enabled V2I, Car-Play- or android-system-enabled V2P, and media-oriented system transport (MOST)-enabled V2S. The access-technology-oriented HIG consists of three major functional modules, including Internetwork Mobility Management (IMM), Network-Specific Authentication and Authorization (NSAA), and Network Traffic Management (NTM). The operational flow and association among key functional modules in the HIG are presented in Figure 6b. 

The IMM module implements mobile IP by utilizing network tunneling between vehicle home agent (VHA) and vehicle foreign agent (VFA). During an on-road journey, the initial operating network enables a home agent, whereas any other network throughout the journey enables a foreign agent for each vehicle. The tunnel-oriented internetwork mobility enabling supports seamless roaming without IP conversion. The NSAA module executes local credential verification for vehicles. It enables HAA to carry out credential verification with the coordination between VFA and VHA. The network traffic management module implements network policies for providing network monitoring services in a particular network. These policies vary with the type of network in heterogeneous network environments. The policy-oriented network monitoring is based on the historical-traffic-usage data and the live traffic data for a specific network connection.

(3) Smart Services as Clients

**Figure 7:**
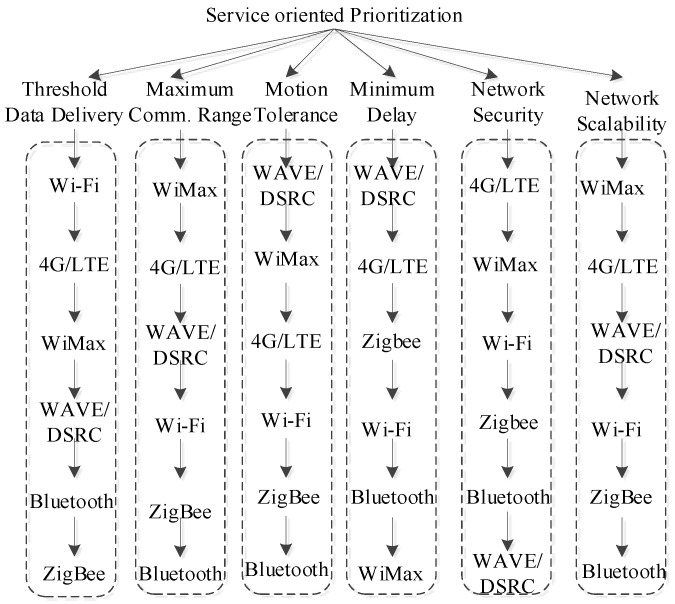
Experimentally validated access-technology prioritization tree.

A client application utilizes access-technology-oriented connection for realizing large cloud-based services in vehicles. Some novel smart client applications are implemented based on cloud service architecture. There are two broader aspects of these client applications in IoV. One is business-focused client applications, majorly oriented toward vehicle insurance, car-sharing, and infotainment. The safety and management-oriented client applications are related to navigation, vehicle diagnostic, and remote telematics in vehicles. Some potential service-oriented clients are materialized below by identifying their parameter-oriented service requirements and corresponding access-technology prioritization (see Figure 7 and Table 2).

- Machine-to-Cloud-Oriented Accident Prevention

The machine-to-cloud (M2C)-oriented traffic safety service in the IoV is implemented considering traffic data inferred knowledge towards accident prevention. It is an advancement in machine-to-machine (M2M)-oriented safety application, majorly relying on locally inferred knowledge from neighboring vehicle’s data. The cloud-server-based smart traffic applications utilize global traffic knowledge to improve decision-making for drivers. It focuses on automatic operations on the go, including steering control, speed control, stoppage, and lane change.

- Black-Box-Oriented Emergency Call Guarantee

The emergency call guarantee service is implemented via heterogeneous network cooperation in the IoV. A vehicle uses the nearest and best available network-access technology to call emergency facilities. The call is forwarded between heterogeneous networks to guarantee its quality and completion for the desired facility. The call implementation considers interval-based manual intimation as well as event-based automatic intimation. The call implementation realizes present and past information-sharing regarding emergency incidence, including speed, direction, location, and lane.

- Mobile Edge Computing–Oriented Parking Helper

The parking helper service is implemented, enabling roadside units as mobile edge computing (MEC). The geographical parking space information dissemination and precise localization are the main technical modules involved in realizing MEC-enabled parking helpers. The implementation is based on Wi-Fi-enabled publish–subscribe communication. The roadside units periodically publish parking availability information. The information is accessed by subscribed passing-by vehicles as receiver-initiated information dissemination.

- Remote-Operation-Oriented Vehicular Telematics

The remote vehicular telematics service is implemented considering guaranteed end-to-end communication between vehicle and remote services. The implementation focuses on executing non-driving operations such as password-oriented vehicle authentication and authorization, intimation of vehicle access, and remote vehicle monitoring. These remote operations exploit heterogeneous vehicular communication to transform the existing physical entity-oriented operations into digital entity-oriented forms.

### 3.3. Network Prioritization in Heterogeneous Vehicular Networks

In the integrated vehicular network environment, the QoS path was computed using a weighted internetwork routing approach. The integrated network path having the greatest weight was chosen as a target for enabling a specific traffic service. The information about the current traffic load was collected from each vehicular communication link using switch port counters traffic history. The concept of multitenancy was used here in order to maximize the QoS requirements in an heterogeneous vehicular network environment. Initially the concept of network virtualization was used to isolate the flow among different vehicular tenants. It was achieved by dividing the vehicular network into layers of local and integrated vehicular networks. Secondly, the weighted internetwork routing algorithm was used to control the allocation of new flows entering into the vehicle network and to prioritize them as per the need of client-specific traffic services. 

A software defined network (SDN) controller was used for vehicular communication flow level network prioritization. The SDN controller was modeled using an undirected graph G, where G=(V,E) is undirected graph among vehicular nodes and existing network infrastructure in traffic environment. Here, V is the set of vehicular nodes in the network and E is the set of vehicular communication links between the network nodes. Considering n as the number of vehicular tenants in the vehicular network graph G, the graph can be divided into various subgraphs including G1, G2, G3,…Gn based on the number of flows and tenants in the traffic-specific network. This graph-centric vehicular network can be represented as expressed in Equation (1).

(1) $$G_{n}=\left(V_{n},\;E_{n}\right),\;\mathrm{where}\;V_{n}\in V,\;\;\mathrm{and}\;E_{n}\in E$$

where $V_{n}$ denotes the set of vehicular nodes as vertices and $E_{n}$ denotes the set of heterogeneous vehicular communication links as edges in the vehicular network subgraph $G_{n}$. Each vehicular node in the subgraph was included in the heterogeneous network G so that all possible combination of heterogeneous vehicular communication paths could be explored by network prioritization component from a source vehicular node to any destination client services of another vehicular node. Each subgraph $G_{n}$ was considered as vehicular nodes with heterogeneous communication links connected to the vehicular graph. Here, two vehicular network subgraphs enabled by different types of local networks were considered edge disjoint. However, it is highlighted that different vehicular communication paths in a subgraph network may have common communication links as common edges. This can be mathematically represented as expressed in Equation (2).

(2) $$G_{n\left(a,b\right)}=\{\begin{array}{c} E_{a}\cap^{\;}E_{b}=0,\;if\;G_{a}\;and\;G_{b}\;are\;different\;types\;of\;vehicular\;networks\;\\ e\in l_{1}\wedge e\in l_{2},\;\;if\;l_{1},\;l_{2}\;are\;from\;same\;network,\;l_{1},\;l_{2}\in G_{a}\;or\;l_{1},\;l_{2}\in G_{b}\; \end{array}$$

where $E_{a}$ and $E_{b}$ denote set of heterogeneous connectivity links as edges of subgraphs $G_{a}$ and $G_{b},$ respectively, and e denotes a common links of the two communication paths $l_{1}$ and $l_{2}$ of same network type. 

The multitenancy QoS prioritization was implemented by using Dijkstra’s and Kruskal’s algorithms for managing the shared vehicular communication resources and their utilization. The shortest heterogeneous path between any two vehicular nodes was computed using the algorithms considering two sets of vehicular network space and heterogenous vehicular communication paths. It is highlighted that the two algorithms were used to carry out heterogeneous vehicular network prioritization for enabling smart traffic client services described in the network architecture. These algorithms were used for two-level network prioritizations. Specifically, in first-level prioritization, Dijkstra’s algorithm was utilized for localized vehicular network without considering other existing network infrastructure nearby the traffic environment. In second-level prioritization, Kruskal’s algorithm was utilized for spanning tree-centric integrated heterogeneous network prioritization where different types of existing networks are considered for enabling vehicular network services. In the two-level prioritization, the shortest heterogeneous vehicular communication path between vehicular nodes was identified considering vehicular flows in the network. The following constraints exist in the two-level prioritization, as expressed in Equations (3) and (4).

(3) $$F\subset V^{2}$$

(4) SP⊂F

where F denotes the set of vehicular node flows between any source and destination, and SP denotes a shortest path between the source and destination vehicular nodes. Here, each heterogeneous communication paths l in the network consists of number of subpaths $P_{l}$ and segments $S_{l}\;$ joining the local vehicular networks. For obtaining the shortest heterogeneous communication path in the shared link resource environment, link utilization ratio $L^{UR}$ of the network was computed as expressed in Equation (5)

(5) $$L^{UR}=\frac{Traffic\;link\;load\;from\;SDN\;switch\;port}{Link\;capacity}=\frac{\lambda_{l}}{C_{l}}$$

where $\lambda_{l}$ denotes the link load of a shared link in a particular path, and $C_{l}$ denotes the link capacity of a shared link in a particular path. The shared link utilization ration $L^{UR}$ was further used in computing the weight $w_{l}$ of a particular path l for making shartest heterogeneous path decsion as expressed in Equation (6).

(6) $$w_{l}=1-\frac{\left(\sum_{m}\sum_{n}L^{UR}\right)/P_{l}}{\sum_{l}\left(\sum_{m}\sum_{n}L^{UR}\right)/P_{l}}\;\mathrm{where}\;w_{l}\epsilon \left(0,1\right)\;\mathrm{and}\;\underset{l}{\sum }w_{l}=1$$

where m and n represents the two vehicular nodes attempting to communicat via heterogeneous vehicular communication links. The path with the highest weight in the heterogenious vehicular network graph was considered the least loaded path and appropriate for establishing prioritized heterogeneous vehicular communication.

## 4. Performance Evaluation—A Case Study

In this section, the performance of service-oriented clients is evaluated in heterogeneous vehicular networking environments as an IoV implementation. Compared with the traditional ad hoc vehicular system, such as VANETs implementation, where infrastructure support is omitted, including RSU, Wi-Fi, and LTE infrastructure.

### 4.1. Simulation Setting and Metrics

**Figure 8:**
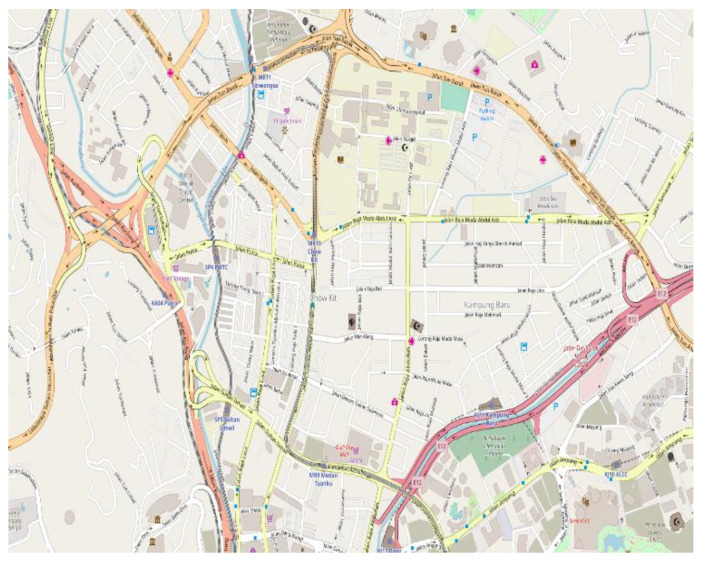
Simulation scenario as open street view.

The heterogeneous networking environment was simulated using network simulator ns-2. A vehicular mobility model generator MOVE and a geographic information system ArcGIS were utilized as supporting applications. Initially, a realistic vehicular mobility network scenario focused on road map editor and vehicle movement editor utility in MOVE. The scenario was executed on a real city road map obtained via a web application open street map (see Figure 8). Two-dimensional location coordinates were precisely embedded on the map using ArcGIS. Notably, the capital city is a real example of dense urban infrastructure where heterogeneous vehicular network environments are a reality. The five types of vehicular communications are implemented, focusing on varying transmission ranges and access technologies. The traditional vehicular communications, including V2V and V2R, were implemented considering IEEE 802.11p-enabled access technology with a [200–300 m] transmission range. The short-range vehicular communications, including V2S and V2P, were implemented considering Wi-Fi-enabled access technology with a lower transmission range, precisely [5–10 m] and [40–80 m] for the respective cases. The more extensive vehicular mobile network was implemented using 4G-enabled access technology considering [500–1000 m]. It is clarified that the tool used to carry out experiments supports 3G and 4G services; therefore, it was mentioned in the paper. However, 5G can be used wherever 4G has been considered. We did not test 5G experimentally in our proposal; therefore, we do not mention it.

**Figure 9:**
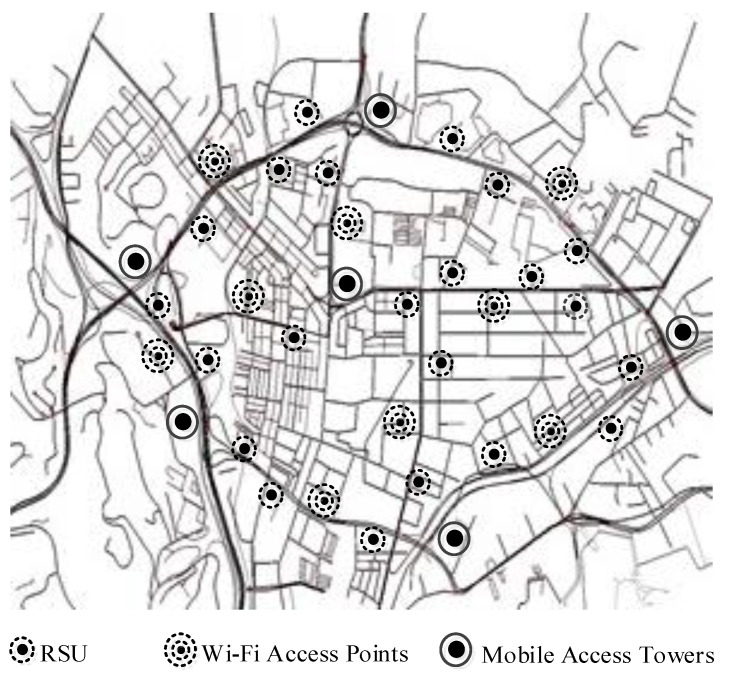
Simulation scenario as simulator view.

The cooperation among these access technologies was realized by considering heterogeneous network access points in the implementation scenario (see Figure 9). The access points included 6 4G-enabled mobile access points at junctions, 9 Wi-Fi-enabled access points, and 22 RSUs alongside roads. Two major traffic scenarios in urban environments were considered in the implementation: peak and off-peak hours’ traffic, where average intervehicle distance and speed are lower and higher, respectively. The values of other general simulation parameters were similar to those considered in vehicular networking implementations [12]. Measuring the performance of the considered IoV clients under these scenarios was attractive for the standardization of heterogeneous vehicular networking and related client implementations. 

The performance of M2C-enabled accident prevention was measured via message diversion rate (i.e., the percentage of vehicles receiving an accident intimation distributed via a dedicated cloud server on the point incidence road section) on the road section of interest. The black-box-oriented emergency call guarantee was measured in message drop rate (i.e., the percentage of emergency messages with a failed delivery attempt in point-to-point short message delivery) in point-to-point emergency message delivery. The MEC-enabled parking helper was evaluated in terms of delay in RSU-enabled distributed message delivery. The performance of remote-operation-oriented vehicular telematics was measured via in-stream utilization in video data delivery. It focused on stream density (i.e., the percentage of neighboring vehicles utilized for establishing multiple-stream-oriented communication path between source and destinations) targeting stream-oriented data delivery in the IoV. It is highlighted that wherever we refer to VANET implementation in the paper, it means a localized vehicular network implementation without considering the heterogeneous existing networks’ integration. We compared a VANET implementation with an IoV implementation, which means an integrated vehicular network environment considering other existing network infrastructures along with the vehicular network.

### 4.2. Analysis of Results

**Figure 10:**
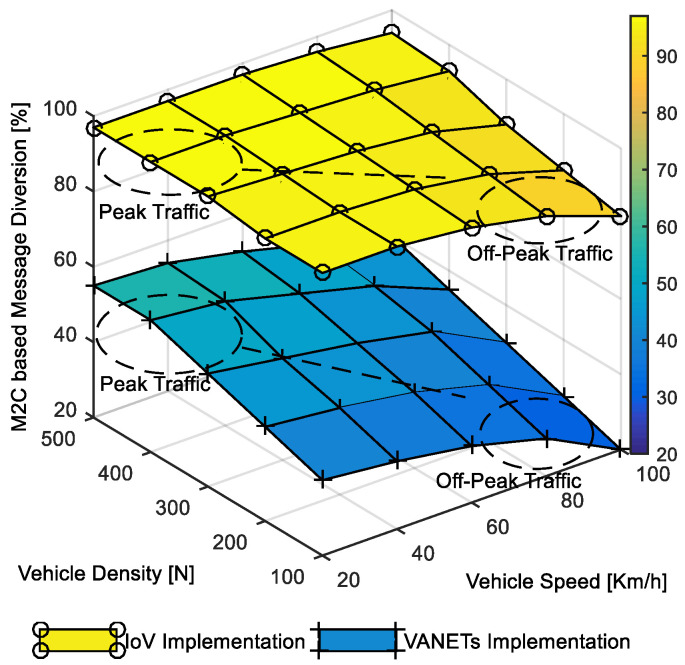
Message diversion in M2C-based accident prevention.

The comparative investigation between IoV and traditional VANETs implementations presented in Figure 10 focuses on-peak and off-peak traffic-based diversion rate evaluation. It can be observed that the message diversion rate was stable at around 90% in the case of IoV implementation. The stability in message diversion can be attributed to the capability of alternative vehicular network selection in the absence of ad hoc vehicular nodes during off-peak traffic. The diversion rate varies with a more extensive range of 20–55% between peak and off-peak traffic in the case of the traditional implementation. The higher variation in diversion rate can be attributed to the availability of vehicular nodes in ad hoc implementation, which is relatively lower during off-peak traffic and higher during peak traffic. The M2C-oriented accident prevention message diversion rate analysis highlighted the benefits of heterogeneous cooperative vehicular networking.

**Figure 11:**
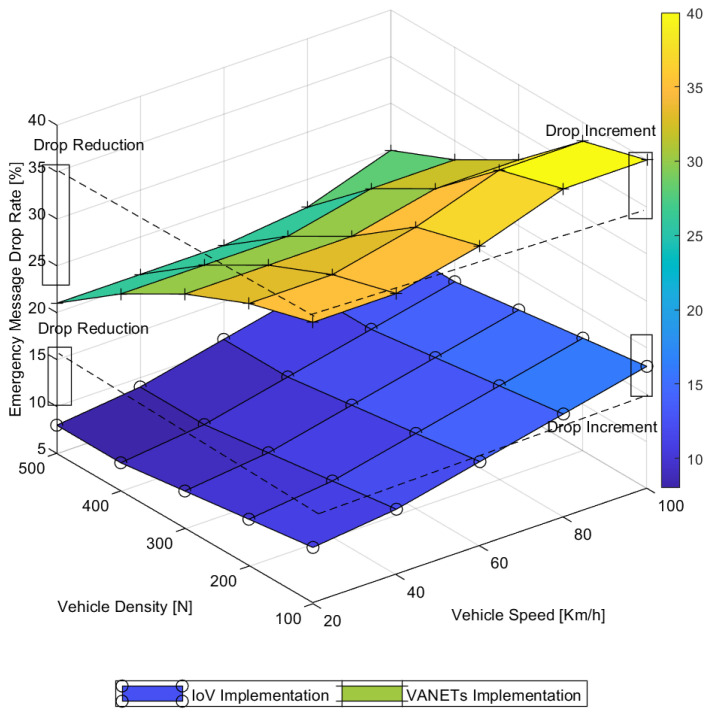
Message drop in back-box-oriented emergency message delivery.

The comparative investigation in Figure 11 focused on drop reduction with higher vehicle density and drop increment with higher vehicle speed. It can be observed that the drop reduction due to vehicle density was relatively higher in the VANET implementation as compared to that in IoV implementations. It reduced from 35% to 20% in the state-of-the-art VANET implementation and from 12% to 9% in the IoV implementation. This can be attributed to the better opportunistic ad hoc networking probability with higher vehicle density for the traditional implementation and better forwarding network selection in the case of the IoV implementation. It is also clearly visible that the drop increment due to higher vehicle speed was approximately equivalent for both the implementations. This was due to the speed-oriented link failure in communication between vehicles and emergency services, which was quite similar in both the implementation scenarios. The emergency message drop analysis highlights the benefit of IoV implementation as an overall lower message drop rate than the traditional implementation. 

**Figure 12:**
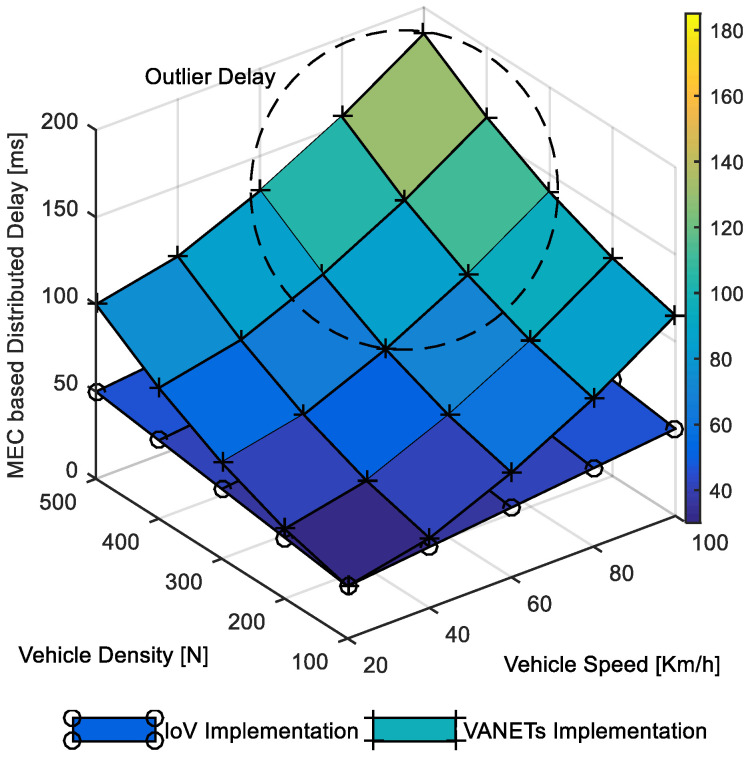
Distributed delay in MEC-based parking helper.

The comparative performance evaluation in Figure 12 focused on outlier delay analysis to enable threshold monitoring for MEC-based clients. It is visible that the impact of speed and density on the distributed operation delay of IoV was comparably negligible. It was in the range of 45–50 ms throughout the density and speed change. This can be attributed to the better operational network availability for distributed operation of clients in heterogeneous IoV environments. The critical impact of higher vehicle speed and density on delay is visible as outlier delay in the case of the VANET implementation. This is an exciting result and provides clear evidence toward poor distributed network management in ad hoc vehicular implementation without considering infrastructure support. The comparative delay analysis for an MEC-based parking helper client attests to the better-distributed network operation capability of the IoV implementation compared to the ad hoc vehicular implementation.

**Figure 13:**
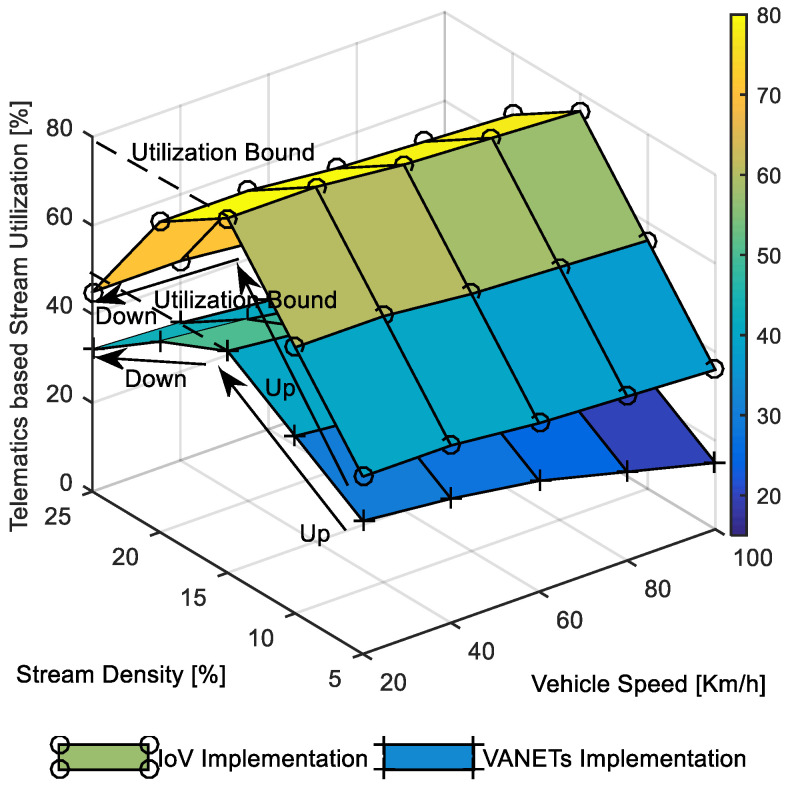
Stream utilization in telematics-based video data delivery.

The comparative performance evaluation presented in Figure 13 focused on monitoring utilization bound. It is visible that the stream utilization bound approximately 80% for IoV implementation case was better than the 45% utilization bound observed in the case of VANET implementation. This can be attributed to the durable stream survivability utilizing heterogeneous links in IoV environments, whereas streams are spontaneous in pure ad hoc implementation environments. It is also noteworthy that the utilization increased upward until stream density reached 12–15%. The stream utilization reduced downward with further higher stream density for both the implementation scenarios. The interesting result shows the particular characteristics of multipath streaming where up to 15% of neighbor node usage for streams supports better network resource utilization. Further usage of neighbor nodes for higher stream density degraded utilization. This was due to the duplication of streams with common neighbor nodes resulting in performance degradation in both the implementation scenarios. 

### 4.3. Summary of Observations

#### 4.3.1. Network Prioritization in Content-Centric Networking

Content-centric networking has significant potential to address the growing heterogeneousness in vehicular environments [21,22]. It effectively reduces point-to-point network load in heterogeneous environments due to its publish/subscribe-based communication architecture. However, content-oriented network prioritization is a challenging task in heterogeneous network environments. 

#### 4.3.2. Virtual Vehicle Hijacking in Vehicular Cyber System

The vehicles are no longer stand-alone entities, particularly with the realization of heterogeneous communication architecture. However, vehicular communication comes with a significant cyber security risk [23]. Specifically, unauthorized wheels access, disabling brakes, locking doors, engine disruption to path forging, location and identity manipulation, and tracking are some examples of virtual vehicle hijacking.

#### 4.3.3. Big Data Analytics in Heterogeneous Traffic Data

The growing heterogeneousness in vehicular traffic data has significantly enlarged traffic data volume towards big data [24]. However, traffic safety and efficiency-oriented intelligent decisions to enable vehicular automation are still based on sensor-based static data. Applying big data analytics in heterogeneous traffic data can bring fundamental changes to the driving experience by inferring sophisticated, intelligent decisions. 

#### 4.3.4. Vehicular-Cooperation-Oriented Edge Computing

The realization of cloud computing in heterogeneous vehicular environments is the need of the hour considering the limited computing capability in distributed vehicular networking [25]. However, enabling cooperation-oriented edge computing can significantly enhance the computing scenario considering the overall growth in on-road vehicles and digital things in vehicular environments. 

#### 4.3.5. Driver Privacy in Heterogeneous Vehicular Communications

Driver privacy is a potential issue due to the growing advancement toward the connected-vehicle environment [26]. There are different types of privacy concerns in connected vehicle environments, including personal information privacy [27], location privacy [28], driving-data privacy [29], third-party privacy [30], and information sharing consent–related privacy [31]. Location privacy has been gaining more attention from researchers in the past few years of connected-vehicle study due to the suitability of location-based communications technologies and services in vehicular traffic environments [32]. Driving data privacy and information sharing are becoming crucial for today’s modern vehicles due to the growing sensor-based technology advancements for connecting vehicles to existing mobile networks and personal gadgets. 

## 5. Conclusions

In this paper, a heterogeneous network model for heterogeneous vehicular communication is presented along with service-oriented implementation. The following conclusions was reached from the design and implementation of the model. The network cooperation enables cloud-oriented computing on big traffic data for realizing intelligent traffic services. The heterogeneous network coordinator and gateway are the key to unambiguous connection management. The service-oriented traffic applications become intelligent with an enlarged traffic data domain and processing capability. The practical simulation verified higher message diversion and stream utilization and lower message drop rate and delays for traffic services in heterogeneous vehicular communication implementation. Mathematical modeling of service-oriented network prioritization and content-centric service implementation in the heterogeneous vehicular environment was also presented to support the heterogeneous vehicular network implemenattion theoretically. In the future, the UAV-enabled networks will be integrated with the heterogeneous vehicular network for enabling specific service-centric real-time vehicular network infrastructure.

## Figures and Tables

**Table 1:** Symbol description.

Symbol	Description
G	Vehicular network connectivity graph
V	Set of vehicular nodes as vertices of the graph
E	Set of vehicular communication links as edges of the graph
F	Set of vehicular communication flows in the network graph
SP	Shortest communication paths between vehicular nodes
S	Number of segments in a particular path l
P	Number of subpaths in a particular path l
$W_{l}$	Weight of a path l used for vehicular path selection
$L^{UR}$	Link utilization ratio of a vehicular network
$\lambda_{l}$	Link load of shared link in a particular path l
$C_{l}$	Link capacity of shared link in aparticular path l

**Table 2:** Client-oriented access technology prioritization.

Client	Client-Oriented Priority Order High 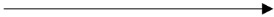 Low
Accident Prevention	WAVE/DSRC → 4G/LTE → ZigBee → Wi-Fi → Bluetooth → WiMax
Emergency Call Guarantee	Bluetooth → ZeeBee → Wi-Fi → WAVE/DSRC → WiMax → 4G/LTE
MEC-Oriented Parking Helper	WiMax → Wi-Fi → 4G/LTE → WAVE/DSRC → Bluetooth → ZigBee
Vehicular Telematics	4G/LTE → WiMax → WAVE/DSRC → Wi-Fi → Bluetooth → ZigBee

## Data Availability

Research data will be made available on individual requests basis to the corresponding author considering research collaboration possibilities with the researchers or research team and with restrictions that the data will be used only for further research in the related literature progress. As the research data will be used by our team for further research in the particular theme.
